# Meta‐analysis: Congruence of genomic and phenotypic differentiation across diverse natural study systems

**DOI:** 10.1111/eva.13264

**Published:** 2021-08-19

**Authors:** Zachary T. Wood, Andrew K. Wiegardt, Kayla L. Barton, Jonathan D. Clark, Jared J. Homola, Brian J. Olsen, Benjamin L. King, Adrienne I. Kovach, Michael T. Kinnison

**Affiliations:** ^1^ School of Biology and Ecology University of Maine Orono ME USA; ^2^ Maine Center for Genetics in the Environment Orono ME USA; ^3^ Department of Natural Resources and the Environment University of New Hampshire Durham NH USA; ^4^ Department of Molecular & Biomedical Sciences University of Maine Orono ME USA; ^5^ Department of Fisheries and Wildlife Michigan State University East Lansing MI USA; ^6^ Department of Wildlife, Fisheries, and Conservation Biology University of Maine Orono ME USA

**Keywords:** candidate gene approaches, *F*_*ST*_, GWAS, natural selection, outlier analysis, *P*_*ST*_

## Abstract

Linking genotype to phenotype is a primary goal for understanding the genomic underpinnings of evolution. However, little work has explored whether patterns of linked genomic and phenotypic differentiation are congruent across natural study systems and traits. Here, we investigate such patterns with a meta‐analysis of studies examining population‐level differentiation at subsets of loci and traits putatively responding to divergent selection. We show that across the 31 studies (88 natural population‐level comparisons) we examined, there was a moderate (*R* ^2^ = 0.39) relationship between genomic differentiation (*F*_*ST*_) and phenotypic differentiation (*P_*ST*_*) for loci and traits putatively under selection. This quantitative relationship between *P*_*ST*_ and *F*_*ST*_ for loci under selection in diverse taxa provides broad context and cross‐system predictions for genomic and phenotypic adaptation by natural selection in natural populations. This context may eventually allow for more precise ideas of what constitutes “strong” differentiation, predictions about the effect size of loci, comparisons of taxa evolving in nonparallel ways, and more. On the other hand, links between *P*_*ST*_ and *F*_*ST*_ within studies were very weak, suggesting that much work remains in linking genomic differentiation to phenotypic differentiation at *specific* phenotypes. We suggest that linking genotypes to specific phenotypes can be improved by correlating genomic and phenotypic differentiation across a spectrum of diverging populations within a taxon and including wide coverage of both genomes and phenomes.

## 1 INTRODUCTION

Quantifying the relationship between genomic and phenomic (Box 1) population differentiation is fundamental to characterizing the genomic basis for phenotypic evolution (Rodríguez‐Verdugo et al., 2017). Understanding the association between genes and phenotypes in natural populations also has the potential to reveal generalizable patterns of evolution (Feder & Mitchell‐Olds, 2003; Gallant & O’Connell, 2020; Rudman et al., 2018). The genomic basis for adaptive evolution also has profound implications for evolutionary conservation, including genetic evolutionary management (Hoffmann et al., 2015; Kinnison & Hairston, 2007) and evolutionary rescue (Carlson et al., 2014). A universal pattern of congruent differentiation in genetic loci and phenotypic traits (i.e., a similar positive relationship between population‐level genomic and phenotypic differentiation for traits and loci putatively under selection) in natural populations would have many theoretical and practical benefits, including context for interspecific comparison of genomic and phenotypic differentiation and generalizable patterns of genomic and phenotypic adaptation. Comparing individual results to generalizable patterns would allow us to address questions such as (1) what constitutes “large” differentiation, (2) whether certain loci have relatively strong effects on phenotypes, and (3) whether nonparallel adaptations are similar in their scope of differentiation, if not in trait pathways.

BOX 1 **What is a phenome?**

Numerous papers use the word *phenome*, a phenotypic analogue to *genome* (Bogue et al., 2018; Burnett et al., 2020; Freimer & Sabatti, 2003; Oti et al., 2008). A genome is the combination of all coding material and corresponding noncoding material in an organism, that is, its DNA or RNA. In theory, a genome can be objectively characterized, though sequencing and alignment choices add a layer of subjectivity to the process. A phenome is the combination of all phenotypic traits of an organism (Oti et al., 2008). Phenotypic traits inherently contain a degree of subjectivity, as we must define phenotypes in order to measure them. For example, a phenotype such as bird wing morphology could be defined by wing size, wing mass, wing shape, number of wing feathers, developmental architecture, and/or other attributes. Some phenotypes, such as behavior, become even more difficult to comprehensively describe. The phenome is also theoretically infinitely large, as measures of phenotypes are constrained in number only by our imagination. One means for limiting the size of the phenome is to consider only ecologically relevant functional traits (ERFTs), or functional traits that affect organismal fitness and interactions with the environment (Wood et al., 2021).

To date, however, the genomic architecture of phenotypic change in most natural populations remains poorly understood, and studies of adaptive population genomics greatly outnumber studies linking genomic change to adaptive phenotypic change (Hendry, 2013, 2016a). Recent technological advances have made sequencing large or whole portions of genomes possible for many nonmodel species (Bolger et al., 2019; Cuperus & Queitsch, 2020; Davey et al., 2011; Goodwin et al., 2016; Russell et al., 2017; Whibley et al., 2021), but are the patterns from these studies generalizable? Specifically, does this growing body of literature support the premise that greater phenotypic differentiation corresponds with greater genomic differentiation in natural organisms (controlling for the number of contributing loci)? Here, we examine this link via standardized measures of genomic differentiation (*F*_*ST*_) and phenotypic differentiation (*P*_*ST*_)—while assessing potential interacting effects associated with different study designs (Box 2). While details of species‐specific genomic architecture certainly affect this link, we sought generalizable patterns at a broader scale, particularly for when information on these specifics is lacking.

BOX 2 ***F*_*ST*_ and *P*_*ST*_**

*F*_*ST*_ is the proportion of genetic variation associated with population structure. As populations become more differentiated, the proportions of alleles at various loci will become less similar across populations, leading to increasing between‐population variation. In principle, *F*_*ST*_ can be calculated as:

$$F_{ST}=\frac{\sum_{j}{\left(p_{j}‐\bar{p}\right)}^{2}N_{j}}{\bar{p}\left(1‐\bar{p}\right)\sum_{j}N_{j}}$$

where *j* represents a population; *p_j_* = the frequency of allele *p* in population *j*; *p*‐bar = the frequency of allele *p* across all populations; and *N_j_* = the number of individuals censused in population *j*.

*P*_*ST*_ is a phenotypic analogue for *F*_*ST*_ and measures the proportion of phenotypic variation associated with population structure. The more differentiated two populations are for a particular phenotype, the greater the proportion of phenotypic variation will be explained by population structure, and the higher *P*_*ST*_ will be. *P*_*ST*_ can be calculated as:

$$P_{ST}=1‐\frac{\sum_{j}\sum_{i}{\left(x_{j_{i}}‐\bar{x_{j}}\right)}^{2}}{\sum_{j}\sum_{i}{\left(x_{j_{i}}‐\bar{x}\right)}^{2}}$$

where the numerator is the sum of squared deviations of each individual's phenotype (*x_ji_*) from the population mean (*x_j_*‐bar) across all populations; and the denominator is the sum of squared deviations of each individual's phenotype (*x_ji_*) from the metapopulation mean (*x*‐bar).

*P*_*ST*_ is analogous to several other common statistics of variance, including *R* ^2^ and *F* ratios. For a model in which the only independent variable is a categorical variable for population:

$$P_{ST}=R^{2}$$

For any model including a variable for population:

$$P_{ST}=\frac{f_{S}\frac{\nu_{n_{S}}}{\nu_{d_{S}}}}{1+\sum_{Z}f_{Z}\frac{\nu_{n_{Z}}}{\nu_{d_{Z}}}}$$

where *f*_S_ = the *F* ratio for the population variable; $\nu_{n_{S}}$ and $\nu_{d_{S}}$ are the numerator and denominator degrees of freedom for the population variable; and *Z* represents all other covariates in the model.

For mean and standard deviation data:

$$P_{ST}=\frac{\sum_{j}s_{j}^{2}N_{j}}{\sum_{j}s_{j}^{2}N_{j}+\sum_{j}{\left(\bar{x_{j}}‐\bar{x}\right)}^{2}N_{j}}$$

where *j* indicates a population; *s_j_* = standard deviation for population *j*; *N_j_* = number of individuals censused in population *j*; and *x_J_*‐bar and *x*‐bar indicate population‐specific and metapopulation means, respectively.

*Q*_*ST*_ is calculated the same way as *P*_*ST*_, but only applies to phenotypes for which the heritable component has been isolated, usually via common‐rearing experiments.

The keystone fact of the Modern Evolutionary Synthesis is the genetic basis for evolution (Fisher, 1930; Huxley, 1942). While phenotypes determine fitness, their heritable, genetic basis controls the response of phenotypes to selection and their persistence in time. In a small but growing number of cases, clear relationships between phenotypes subject to natural selection and their associated genes (e.g., Barrett et al., 2019; Colosimo et al., 2004) have been identified in natural populations. However, the ability to associate genetic variation with phenotypes in natural populations—where environmental conditions are beyond manipulation—remains challenging (Hendry, 2013, 2016a). Nonetheless, substantial progress in linking genetic and phenotypic variation has been made in limited cases (i.e., genome‐wide association studies; GWAS: Visscher et al., 2017).

Despite this progress, biologists have struggled to systematically associate genomic data with biologically relevant phenotypes, particularly when pleiotropy, polygenic inheritance, epistasis, and phenotypic plasticity confound their relationship (Pigliucci & Muller, 2010; Walsh & Lynch, 2018). In a large proportion of studies of adaptation in natural populations, genomic variation is analyzed for signals of selection without any direct quantification of biologically relevant phenotypic trait variation. As such, most literature on the heritable basis of adaptation tends to focus primarily on the characterization of either genomic or phenotypic variation in natural populations, but not explicitly link the two. Genome‐wide association studies (GWAS) provide one avenue to explore genotype–phenotype relationships in natural populations, but are often plagued by high false‐positive rates and commonly struggle to detect the small genetic effect sizes of many polygenic traits (Chen et al., 2021; Evangelou & Ioannidis, 2013). This growing body of studies has attempted to associate genomic and phenotypic aspects of adaptation in the same diverging populations of organisms. These studies in turn provide a means to assess how genomic and phenotypic variation are distributed among populations experiencing ongoing adaptive differentiation. Genetic and phenotypic differentiation are particularly useful for linking genotypes to phenotypes, as they produce phenotypic and genetic variation, which can then be harnessed statistically for GWAS or outlier studies (Gibson, 2018; Visscher & Goddard, 2019).

While numerous phenotypic traits clearly have a heritable basis, their underlying genomic architecture is rarely fully—or even mostly—explained, leading to what is sometimes called the “missing heritability problem” (Young, 2019; Zuk et al., 2012). This is not entirely unexpected given the great complexity of genomes and phenomes, and the constraints both present for statistical power (López‐Cortegano & Caballero, 2019; Uricchio, 2020). This recognized challenge has led to substantial innovation—and thus variability—among investigators and studies in methods used to associate genomic and phenotypic differentiation (Burt & Munafò, 2021). Despite a growing number of approaches, no clear best practices exist for linking genotype to phenotype across systems. Each method has substantial limitations, and the lack of best practices adds noise to any attempt to detect underlying trends common across the tree of life (Tam et al., 2019).

For example, while gene‐knockout experiments provide an ideal means of studying how variation in a particular candidate gene determines phenotype when the species can be reared in a laboratory setting (Hall et al., 2009), these experiments are prohibitive or unethical for studies of most nonmodel species in natural systems. Further key choices in study system, study design, genomic data collection, and analytical approach all likely influence calculations of genomic and phenotypic differentiation in idiosyncratic ways. Controlling for methodological variation can therefore potentially reveal more generalizable patterns that may help in understanding the relationship between genomic and phenotypic differentiation, allowing for cross‐system comparisons and generalizations about responses of natural populations to selection.

Here, we conduct a meta‐analysis of 31 studies of natural populations representing 88 unique multi‐population comparisons that demonstrate putative genomic and phenotypic differentiation in response to selection. We address two main questions:

*How does genomic differentiation at loci under selection explain phenotypic differentiation, both across and within studies?* Under ideal conditions, when all the loci underlying a phenotypic trait are identified and the phenotype is accurately quantified, we would expect a strong, positive relationship between *P*_*ST*_ and *F*_*ST*_ for loci under selection (Brommer, 2011; Kaeuffer et al., 2012; Raeymaekers et al., 2007). However, measuring numerous genotypes and phenotypes inherently leaves much room for error, even beyond methodological nuances, as not all highly differentiated loci will code for highly differentiated phenotypes, and some important loci may exhibit little differentiation, muddying the relationship between *P*_*ST*_ and *F*_*ST*_. Fundamental differences in genomic architecture—including the strength of individual loci (many weak vs. few strong), linkage, and gene interactions—across taxa and traits will also obscure the relationship between *P*_*ST*_ and *F*_*ST*_ (Keane et al., 2011). Finally, some phenotypic differentiation will simply be explained by neutral genomic differentiation (Raeymaekers et al., 2017; Whitlock, 2008; Zhang, 2018).

*How do key methodological choices affect the strength of the genome‐to‐phenome association?* Differences in methodological choices for genome‐to‐phenome studies are likely to affect not only conclusions about the extent of genomic and phenotypic differentiation, but the expected relationship between *P*_*ST*_ and *F*_*ST*_ as well. As some genomic markers are more likely to fall in or near coding or modifier regions (Box 3), those markers may have stronger relationships with phenotypes. Furthermore, smoothing or adjusting *F*_*ST*_ and correcting for false‐positive rates may improve statistical error rates, but bias the relationships between *P*_*ST*_ and *F*_*ST*_, in part by changing the proportion of the genome characterized as “non‐neutral” (Lotterhos & Whitlock, 2014; Luu et al., 2017). Finally, common‐rearing experiments may avoid some of these challenges by isolating genetic differences in phenotypes, but they also remove gene‐by‐environment interactions, which are important genetically based sources of phenotypic variation (Via & Lande, 1985).

BOX 3 **Common genomic markers**

Over the past several decades, genetic variation has been assayed using a variety of molecular markers. Rapid advancements in DNA sequencing technologies now allow researchers to cost‐effectively sequence whole or large proportions of genomes. The following markers are frequently used to study genomic differentiation.

**Microsatellites (msats)**

Msats are short (typically 2–4 nucleotide) sequence repeats (typically 5–50 times) of DNA in noncoding regions that vary in length between individuals and thus are generally regarded as neutrally evolving loci (Schlötterer, 2000). Msats are also referred to as short tandem repeats (STRs) and simple sequence repeats (SSRs). Occasionally msats demonstrate signals of selection, likely due to linkage with coding regions experiencing a selective sweep (i.e., genetic hitchhiking). However, in rare cases, msats may be directly involved in phenotype determination by affecting gene expression (Li et al., 2002) or when they occur within a gene (Li et al., 2004).

**Amplified fragment length polymorphisms (AFLPs)**

AFLP methods assess the fragment profile of DNA that has been amplified after digestion with restriction enzymes. AFLPs provide biallelic genotypes based on presence/absence scoring. Hundreds of loci can be assayed for relatively little cost, making ALFPs a useful tool for studying patterns of selection across the genome without any knowledge of the genome sequence.

**Single nucleotide polymorphisms (SNPs)**

A SNP is DNA variation that occurs at a single nucleotide position. While most SNPs have no effect on fitness because either they are in neutral regions of the genome or represent a silent mutation (i.e., one that does not affect amino acid coding), some SNPs in coding regions can be highly influential. Sliding windows can be used to measure the collective *F*_ST_ of numerous nearby SNPs, thus isolating islands of genomic differentiation, rather than single differentiated SNPs.

**Quantitative trait loci (QTLs)**

QTLs are genomic regions that are significantly associated with a quantitative, or continuous trait with a polygenic basis. These regions are defined using experimental crosses in a process called QTL mapping (Sen & Churchill, 2001). Regions are genotyped using msats or SNP markers.

**Haplotypes/Microhaplotypes**

Most SNP genotyping methods involve sequencing contiguous sets of nucleotides that may contain multiple polymorphic sites. A microhaplotype genotyping approach considers all the neighboring SNPs present on a single sequencing read to be representative of single allele/haplotype due to the assumption of extremely low recombination rates over short distances (<300 nucleotides). With this approach, a locus that contains multiple SNPs is then analyzed in a multiallelic framework, much like a microsatellite.

## 2 METHODS

### 2.1 Overview

We used Web of Science (https://webofknowledge.com) and the search terms in Table S1 to isolate 88 population‐level comparisons that included phenotypic and genotypic data from two or more populations under purported divergent selection (Table 1). We extracted or calculated three metrics from each paper for all possible pairwise comparisons of each phenotype measured between populations: (1) *P*_*ST*_, phenotypic differentiation, (2) neutral *F*_*ST*_, neutral genomic differentiation, and (3) non‐neutral *F*_*ST*_ (henceforth: *nnF*_*ST*_), genetic differentiation for loci putatively under selection (i.e., candidate genes, outlier loci, or loci associated with a differentiated phenotype in a GWAS) (Wright, 1949). We also included methodological covariates, including the method of determining loci under selection (Box 4), type of genetic marker used (Box 3), the software used to calculate *F*_*ST*_, whether the study included a common‐garden design, and the proportion of loci identified as non‐neutral.

**TABLE 1:** Papers used in this meta‐analysis

Paper^a^	Species	Method^b^	Phens.^c^	Unique comps.^d^	Mean N^e^	Marker type	Analysis methods^f^	Common garden
Defaveri & Merilä (2013)	*Gasterosteus aculeatus*	C	1	1	34.8	QTL	R	No
Morris et al. (2018)	*Gasterosteus aculeatus*	C	4	1	39.2	QTL	R	No
Paccard et al. (2018)	*Gasterosteus aculeatus*	C	6	1	25.4	MSAT	R	No
Le Corre (2005)	*Arabidopsis thaliana*	C	12	1	NA	Haplo.	R	Yes
Pedersen et al. (2017)	*Gasterosteus aculeatus*	C, O	2	1	116.8	SNP	N, R	No
Ólafsdóttir & Snorrason (2009)	*Gasterosteus aculeatus*	C, O	3	1	46.0	MSAT	N, R	No
Royer et al. (2016)	*Yucca* spp	G	3	1	103.0	SNP	G	No
Johnston et al. (2014)	*Salmo salar*	G, O	1	1	125.8	SNP	N, B	No
Wei et al. (2017)	*Brassica napus*	G, O	4	2	108.6	SNP, Window	G	Yes
Porth et al. (2015)	*Populus trichocarpa*	G, O	113	1	108.3	SNP	N, B	No
Laporte et al. (2015)*	*Coregonus clupeaformis*	O	1	5	30.0	SNP	N	No
Marques et al. (2017)	*Gasterosteus aculeatus*	O	1	1	70.0	SNP	N	No
Raeymaekers et al. (2007)*	*Gasterosteus aculeatus*	O	1	6	30.0	QTL	N	No
Kovi et al. (2015)	*Lolium perenne*	O	1	1	300.0	SNP	N	Yes
Izuno et al. (2017)	*Metrosideros polymorpha*	O	2	1	8.0	SNP	B	Yes
Smith et al. (2008)	*Andropadus virens*	O	3	1	20.9	AFLP	N	No
Qiu et al. (2017)	*Phragmites australis*	O	3	1	9.0	AFLP	N, B	Yes
He et al. (2019)	*Banksia attenuata*	O	4	1	11.0	SNP	B	No
Sra et al. (2019)	*Brassica* spp	O	4	3	270.3	SNP	N	Yes
Hamlin & Arnold (2015)*	*Iris hexagona*	O	5	22	10.1	SNP	B	No
Nakazato et al. (2012)	*Solanum peruvianum*	O	5	1	10.9	AFLP	N	Yes
Hudson et al. (2013)*	*Coregonus* spp	O	6	9	6.8	AFLP	N, B	No
Kaeuffer et al. (2012)*	*Gasterosteus aculeatus*	O	9	6	40.0	MSAT	R	No
Culling et al. (2013)*	*Salmo salar*	O	9	10	36.8	SNP	N, B	Yes
Sedeek et al. (2014)	*Ophrys* spp	O	10	1	28.4	SNP	B	No
Keller et al. (2011)	*Populus balsamifera*	O	10	3	15.2	SNP	N	Yes
N’Diaye et al. (2018)	*Triticum turgidum*	O	11	1	42.7	SNP	N, B	Yes
Eimanifar et al. (2018)	*Apis mellifera*	O	13	1	154.7	SNP	N	No
Porth et al. (2016)	*Quercus* spp	O	13	1	827.5	SNP	R	No
Flanagan et al. (2016)	*Syngnathus scovelli*	O	14	1	21.2	SNP	R	No
Dillon et al. (2013)	*Pinus radiata*	O	39	1	149	SNP	N	Yes

^a^ *Indicates papers in within‐study analysis.

^b^ C = candidate gene; G = GWAS; O = outlier.

^c^ Number of phenotypes studied.

^d^ Unique interpopulation comparisons.

^e^ Mean individuals per population.

^f^ R = raw; N = non‐Bayesian; B = Bayesian; G = GWAS‐specific.

BOX 4 **Genomic methods for detecting divergent selection**

Techniques used for detecting selection can broadly be split into complementary approaches that detect either loci of presumably large effect or those that identify patterns of polygenic selection.

**Candidate gene approaches**

Candidate gene approaches investigate associations between genotypes and phenotypes at specific preordained loci with *a priori* hypothesized functions. Candidate gene approaches are most commonly pursued in species with relatively rich genomic resources. Reproducibility has been challenging for many candidate locus approaches (Tabor et al., 2002).

**Outlier approaches**

Genetic differentiation (e.g., *F*_*ST*_) outlier tests are most commonly used to identify candidate loci. These tests assume that loci with genetic differentiation values that significantly exceed background (i.e., neutral) differentiation levels are under selection.

**Genome‐wide association studies (GWAS)**

Genome‐wide association studies (GWAS) are one suite of methods that identify correlations between genomic data—usually high coverage genomic data—and fitness values, phenotypes, or environmental values (GEAS).

We used general linear models to examine the relationship between phenotypic differentiation (*P*_*ST*_) and (1) neutral and non‐neutral genetic differentiation (*F*_*ST*_, *nnF*_*ST*_), (2) proportion of loci identified as non‐neutral, and (3) several methodological choices. The result is several models that assess the degree to which phenotypic and genomic differentiation are congruent (in traits and loci putatively under selection), as well as the role of some potential confounding methodological factors.

### 2.2 The database

We used all databases within the online citation database Web of Science and the 25 search terms in Table S1 to find relevant papers that included phenotypic and genomic data from two or more populations undergoing divergent selection. Searches returned anywhere from 0 to 340,088 papers; we retained papers revealed by searches with <700 results (Table S1). We used R version 3.6.1 (R Core Team, 2019) and the packages *metagear* (Lajeunesse, 2016) and *Bibliometrix* (Aria & Cuccurullo, 2017) to screen the abstracts of each paper to determine whether the paper was likely to contain both genomic and phenotypic comparisons for multiple populations. For consistency, the same observer (ZTW) reviewed every abstract. To examine how well our search terms captured the breadth of the relevant literature, we conducted a forward–backward literature search following Koricheva et al. (2013). We examined literature cited by and literature which cited every study included in our meta‐analysis for relevance based on the title alone. We used Google Scholar on December 8, 2019, to find literature that cited the papers included in our analysis. We then determined how many of those papers were already captured by our original search terms. Any papers that were not included in our original screening process were then screened based on the abstract as described above; none of the additionally screened papers contained appropriate data for inclusion in our meta‐analysis. In total, we screened 4317 papers, retaining 31 papers for analysis (Figure S1). The most common reason for noninclusion of papers (nearly all) was lack of measured phenotypic data. As these data were generally not measured, rather than not reported, we did not request data from authors.

We extracted the following information from each paper: species, phenotypic trait, *P*_*ST*_ of the phenotypic trait, number of individuals used to calculate *P*_*ST*_, number of groups the phenotypes were sampled from, number of groups the genotype data were sampled from, *F*_*ST*_ of loci under selection (“non‐neutral”; *nnF*_*ST*_), *F*_*ST*_ of neutral loci, *F*_*ST*_ formula, marker type, number of loci under selection, number of neutral loci, and method(s) used to determine which loci are under selection. For papers which included raw phenotypic measurements (16) we calculated *P*_*ST*_ using:

(1) $$P_{\mathrm{ST}}=1-\frac{\sum_{j}\sum_{i}{\left(x_{j_{i}}-\bar{x_{j}}\right)}^{2}}{\sum_{j}\sum_{i}{\left(x_{j_{i}}-\bar{x}\right)}^{2}}$$

The numerator is the sum of squared deviations of each individual's phenotype from the population mean, and the denominator is the sum of squared deviations of each individual's phenotype from the metapopulation mean. We used *F* tests to confirm that all reported and calculated *P*_*ST*_ values were statistically different than 0, that is, implied phenotypic differentiation.

For papers with more than two study populations, we used all possible pairwise population comparisons for analysis.

We organized genomic analysis software into four groups:

1. Raw: raw *F*_*ST*_ calculations
2. Bayesian: BayeScan and 2DSFS
3. Non‐Bayesian: Arlequin, Lositan, fdist, dfdist, fstat, detsel, lnRV
4. GWAS‐specific: other software used specifically for GWAS

### 2.3 Analyses

**FIGURE 1:**
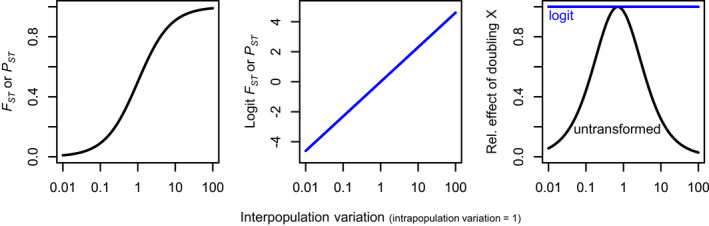
Logit transformations are useful for quantifying differentiation. *Left*: *F*_*ST*_ and *P*_*ST*_, untransformed, provide limited characterization of differentiation when differentiation is low or high (i.e., interpopulation variation is very small or large relative to intrapopulation variation). *Center*: logit transformations of *F*_*ST*_ and *P*_*ST*_, however, provide a log‐linear metric of differentiation whose shape is independent of differentiation. *Right*: doubling differentiation (interpopulation variation) has the same effect on logit(*F*_*ST*_ and *P*_*ST*_) regardless of starting point, whereas the effect of doubling differentiation on untransformed *F*_*ST*_ and *P*_*ST*_ depends heavily on starting point

We logit‐transformed all *F*_*ST*_ and *P*_*ST*_ data in this study. The logit transformation assumes that small changes at the ends of the range of possibilities (e.g., from *F*_*ST*_ = 0.01 to 0.02 or 0.98 to 0.99) are more important than small changes in the middle of the range (e.g., from *F*_*ST*_ = 0.50 to 0.51). Logit transforming *F*_*ST*_ and *P*_*ST*_ also standardizes their sensitivity to differentiation—doubling interpopulation variation produces an equal change in logit(*F*_*ST*_ or *P*_*ST*_) regardless of the starting variation (Figure 1). Finally, logit‐transforming *F*_*ST*_ makes *F*_*ST*_ data—which are often right‐skewed—roughly normally distributed (Figure S2). All neutral *F*_*ST*_ values <0.005 were changed to 0.005 to avoid having zero or negative values, which cannot be handled by a logit transformation.

Due to the large range in number of phenotypes measured in any given paper, we used paper‐specific averages for our global *P*_*ST*_‐*F*_*ST*_ analysis. Specifically, we averaged values for all numeric variables for each unique population‐level comparison, allowing multiple points if a comparison was replicated with multiple methods (i.e., different statistical software or a GWAS and outlier approach). This averaging resulted in a final 111 datapoints for 88 unique population‐level comparisons across 31 papers.

**FIGURE 2:**
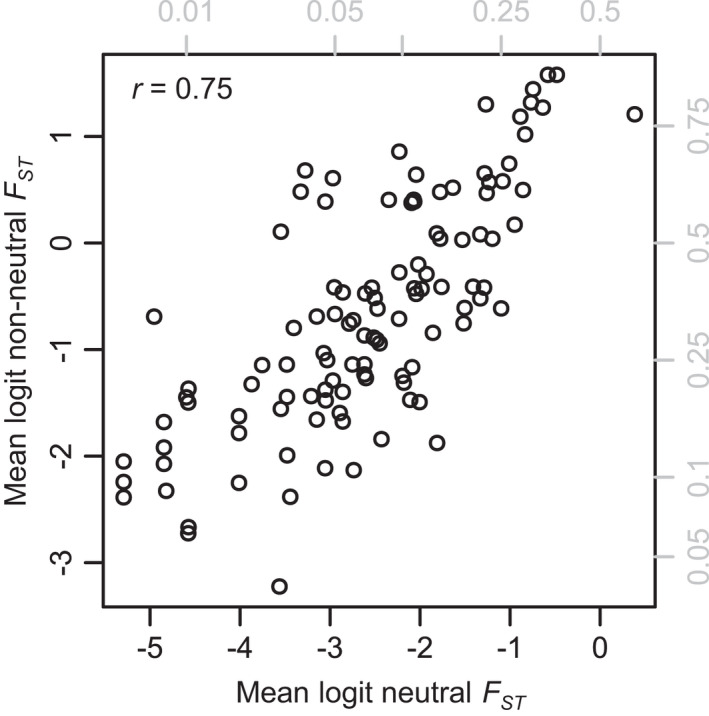
Non‐neutral and neutral *F*_*ST*_ are highly correlated across studies. Gray labels show untransformed *F*_*ST*_ values. Each point represents average *F*_*ST*_ values for a unique population–population comparison, with multiple points for multiple methods (see text)

We then tested these data for relationships between average *P*_*ST*_ and average neutral *F*_*ST*_ or average non‐neutral *F*_*ST*_ (*nnF*_*ST*_). As *nnF*_*ST*_ and *F*_*ST*_ are strongly correlated (Figure 2), including both in the same model is inadvisable; we therefore tested them separately using two general linear models and likelihood ratio tests (Fox & Weisberg, 2011). We compared the models using relative likelihood—as these models were not nested, a likelihood ratio test was not feasible (Burnham & Anderson, 2003).

**FIGURE 3:**
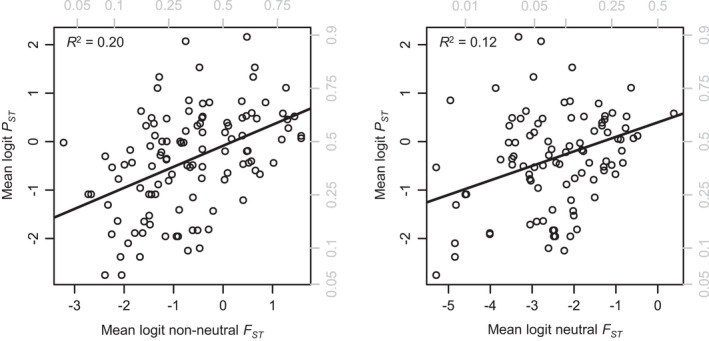
Both non‐neutral (*left*) and neutral (*right*) *F*_*ST*_ predict *P*_*ST*_, but non‐neutral *F*_*ST*_ is a much stronger predictor of *P*_*ST*_. Gray labels show untransformed *F*_*ST*_ and *P* *_ST_* values. Each point represents average *P*_*ST*_ and *F*_*ST*_ values for a unique population–population comparison, with multiple points for multiple methods (see text)

As *nnF*_*ST*_ was superior to neutral *F*_*ST*_ in predicting *P*_*ST*_ (Figure 3), we investigated the proportion of non‐neutral loci as a covariate in the *P*_*ST*_‐*nnF*_*ST*_ relationship. A study that found one locus with high *F*_*ST*_ has different implications for *P*_*ST*_ than a similar study finding hundreds of loci with high *F*_*ST*_. We therefore fit the following general linear model:

(2) $$\text{logit}\left(P_{ST}\right)=\beta_{0}+\beta_{F}\text{logit}\left(nnF_{ST}\right)+\beta_{L}\text{logit}\left(\frac{N_{nn}}{N_{total}}\right)+\beta_{FL}\text{logit}\left(nnF_{ST}\right)\text{logit}\left(\frac{N_{nn}}{N_{total}}\right)$$

*P*_*ST*_ and *nnF*_*ST*_ are described above; *N*_*nn*_ = number of non‐neutral loci; *N*_*total*_ = total number of loci examined; and *β*‐terms are coefficients determined during model fitting.

We tested all effects in the above model using type II likelihood ratio tests. As only the first‐order effect of proportion non‐neutral loci was significant (see Results), we removed the interaction and refit the model. We also included a first‐order effect of proportion of non‐neutral loci in all subsequent analyses.

We tested for the effects of four methodological variables—common‐garden rearing, genetic marker type, broad methodological approach (candidate gene, outlier, GWAS), and analytical method (i.e., software choice)—on *P*_*ST*_ and the *P*_*ST*_‐*nnF*_*ST*_ slope. We did not test for an effect of *nnF*_*ST*_ *p*‐value threshold, as *p*‐value threshold did not have a strong effect on *nnF*_*ST*_ (Figure S3). We tested each of the methodological variables separately to avoid overfitting, as there were only 31 papers in our dataset. We fit the following general linear model for each methodological variable:

(3) $$\text{logit(}P_{ST})=\beta_{M}+\beta_{FM}\text{logit(}nnF_{ST})+\beta_{L}\text{logit}\left(\frac{N_{nn}}{N_{total}}\right)$$

*P*_*ST*_, *nnF*_*ST*_, *N*_*nn*_, and *N*_*total*_ are described above; *β*‐terms are coefficients determined during model fitting: *β*_*M*_ indicates a method‐specific intercept and *β*_*FM*_ indicates a method‐specific *P*_*ST*_‐*nnF*_*ST*_ slope.

We tested all effects in each model using type II likelihood ratio tests.

We also examined *P*_*ST*_‐*nnF*_*ST*_ trends within studies, with the goal of elucidating a *P*_*ST*_‐*nnF*_*ST*_ relationship for individual phenotypes across numerous populations. We winnowed our master database down to all paper‐phenotype‐method combinations that had at least five *P*_*ST*_‐*nnF*_*ST*_ datapoints (18 paper‐phenotype‐method combinations total). We then fit the following general linear model across all datapoints from the winnowed database:

(4) $$\text{logit}\left(P_{ST}\right)=\beta_{Z}+\beta_{FZ}\text{logit}\left(nnF_{ST}\right)+\beta_{L}\text{logit}\left(\frac{N_{nn}}{N_{total}}\right)$$

*P*_*ST*_, *nnF*_*ST*_, *N*_*nn*_, and *N*_*total*_ are described above; *β*‐terms are coefficients determined during model fitting: *β*_*Z*_ indicates a phenotype‐specific intercept and *β*_*FZ*_ indicates a phenotype‐specific *P*_*ST*_‐*nnF*_*ST*_ slope—that is, *β*_*Z*_ and *β*_*FZ*_ took a unique value for each paper‐phenotype‐method combination.

We fit one *β*_*L*_ (proportion of non‐neutral loci) slope across all paper‐phenotype‐method combinations, rather than fitting a unique *β*_*L*_ term for each (as we did for *β*_*Z*_) due to the overall small sample size and the lack of variation in proportion of non‐neutral loci for three papers. We tested the slopes of each phenotype‐specific *P*_*ST*_‐*nnF*_*ST*_ relationship (*β*_*FZ*_) using *t* tests. As the relatively small number of points within each study certainly lowered the power to detect significant *P*_*ST*_‐*nnF*_*ST*_ relationships, we also examined the distribution of *P*_*ST*_‐*nnF*_*ST*_ slopes for the within‐study analysis.

## 3 RESULTS

Non‐neutral *F*_*ST*_ (*nnF*_*ST*_) was superior to neutral *F*_*ST*_ in predicting *P*_*ST*_, with higher slope (0.43 vs. 0.30) and *R* ^2^ (0.20 vs. 0.12) (Figure 3). Both had statistically significant relationships with *P*_*ST*_ (likelihood ratio test—non‐neutral *F*_*ST*_: *χ* ^2^ = 27.3; df = 1; *p* < 0.001. Likelihood ratio test—neutral *F*_*ST*_: *χ* ^2^ = 15.0; df = 1; *p* < 0.001). However, the *nnF*_*ST*_ model outperformed the neutral *F*_*ST*_ model, with the neutral model having a likelihood of 0.005 with respect to the *nnF*_*ST*_ model.

**FIGURE 4:**
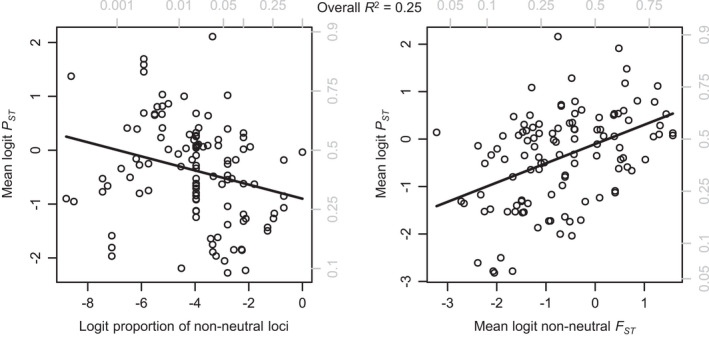
*Left*: proportion of non‐neutral loci (the ratio of candidate, outlier, or GWAS positive loci to the total number examined) has a negative effect on *P*_*ST*_. Removing this effect allows for a clearer view of the *P*_*ST*_‐non‐neutral *F*_*ST*_ relationship (*right*). There is no significant interaction between proportion of non‐neutral loci and non‐neutral *F*_*ST*_ (Table 2). Gray labels show untransformed *F*_*ST*_, *P* *_ST_*, and proportion of non‐neutral loci values. Each point represents average *P*_*ST*_,*F*_*ST*_, and proportion of non‐neutral loci for a unique population–population comparison, with multiple points for multiple methods (see text)

Proportion of non‐neutral loci also had a significant negative effect on *P*_*ST*_, but had no significant interaction with *nnF*_*ST*_ (Figure 4; Table 2). Including proportion of non‐neutral loci in a model with *nnF*_*ST*_ (without the nonsignificant interaction) increased the model *R* ^2^ from 0.20 to 0.25.

**TABLE 2:** Type II likelihood ratio tests for model predicting *P*_*ST*_

Variable	*χ* ^2^	df	*p*
logit(non‐neutral proportion of loci)	7.2	1	0.007
logit(non‐neutral *F*_*ST*_)	24.8	1	<0.001
logit(proportion of loci) × logit(non‐neutral *F*_*ST*_)	0.9	1	0.338

**FIGURE 5:**
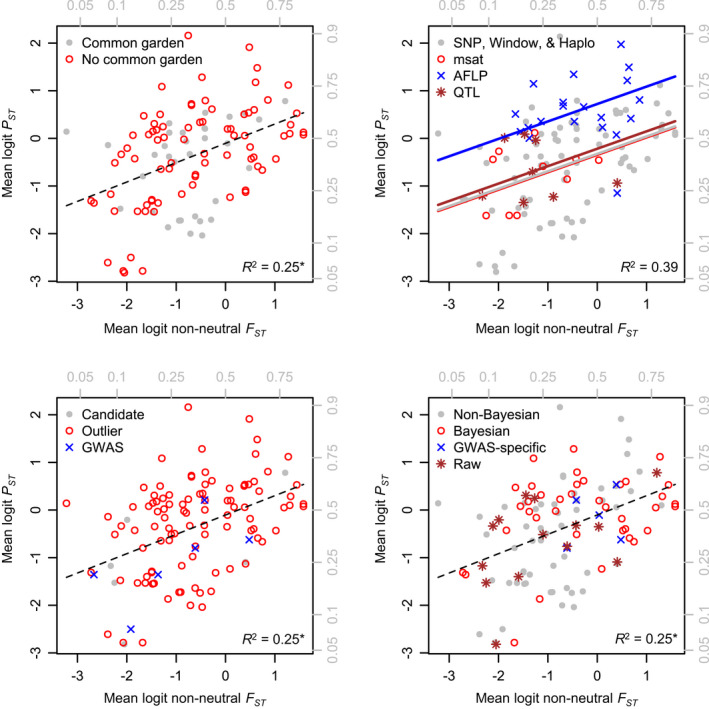
Effects of common‐garden experimentation (*top left*), marker type (*top right*), broad method (*bottom left*), and analysis method (*bottom right*) on *P*_*ST*_ and the *P*_*ST*_‐non‐neutral *F*_*ST*_ slope. Only marker type had a significant effect on *P*_ST_, and none of the four variables had a significant effect on the *P*_*ST*_‐non‐neutral *F*_*ST*_ slope (Table 2). Gray labels show untransformed *F*_*ST*_ and *P* *_ST_* values. Each point represents average *P*_*ST*_ and *F*_*ST*_ values for a unique population–population comparison, with multiple points for multiple methods (see text). Variation due to proportion of non‐neutral loci is removed in each panel. *Only marker type (*top right*) had a significant effect on *P*_*ST*_; *R* ^2^ values and trendlines for the other three models are from the base (Figure 4) model

Of our four methodological variables (common‐garden rearing, genetic marker type, broad method, and software analysis method), only marker type had a significant effect on *P_ST_* (Figure 5; Table 3). *P*_*ST*_ was highest (for any given value of non‐neutral *F*_*ST*_) for AFLPs, then QTLs, which were closely followed by SNPs and msats. This model result does not, however, imply that some marker types *caused* higher *P*_*ST*_, rather it indicates that *P*_*ST*_ was higher for a given estimate of *nnF*_*ST*_ (or more intuitively, *nnF*_*ST*_ was estimated as lower for a given value of *P*_*ST*_) for some markers. Marker type did not have a significant interaction with *nnF*_*ST*_.

**TABLE 3:** Type II likelihood ratio tests for effects of methodological choices on *P*_*ST*_ and the *P*_*ST*_‐*F*_*ST*_ slope

Variable	*χ* ^2^	df	*p*
Common‐garden model			
Common garden	1.3	1	0.260
logit(non‐neutral *F*_*ST*_)	24.6	1	<0.001
Common garden × logit(non‐neutral *F*_*ST*_)	1.3	1	0.251
logit(non‐neutral proportion of loci)	6.1	1	0.014
Marker model			
Marker	24.0	3	<0.001
logit(non‐neutral *F*_*ST*_)	22.6	1	<0.001
Marker × logit(non‐neutral *F*_*ST*_)	2.9	3	0.406
logit(non‐neutral proportion of loci)	3.0	1	0.083
Method model			
Method	4.1	2	0.129
logit(non‐neutral *F*_*ST*_)	20.6	1	<0.001
Method × logit(non‐neutral *F*_*ST*_)	0.3	2	0.849
logit(non‐neutral proportion of loci)	8.9	1	0.003
Analytical method model			
Analytical method	2.3	3	0.521
logit(non‐neutral *F*_*ST*_)	19.6	1	<0.001
Analytical method × logit(non‐neutral *F*_*ST*_)	4.4	3	0.222
logit(non‐neutral proportion of loci)	1.5	1	0.224

**FIGURE 6:**
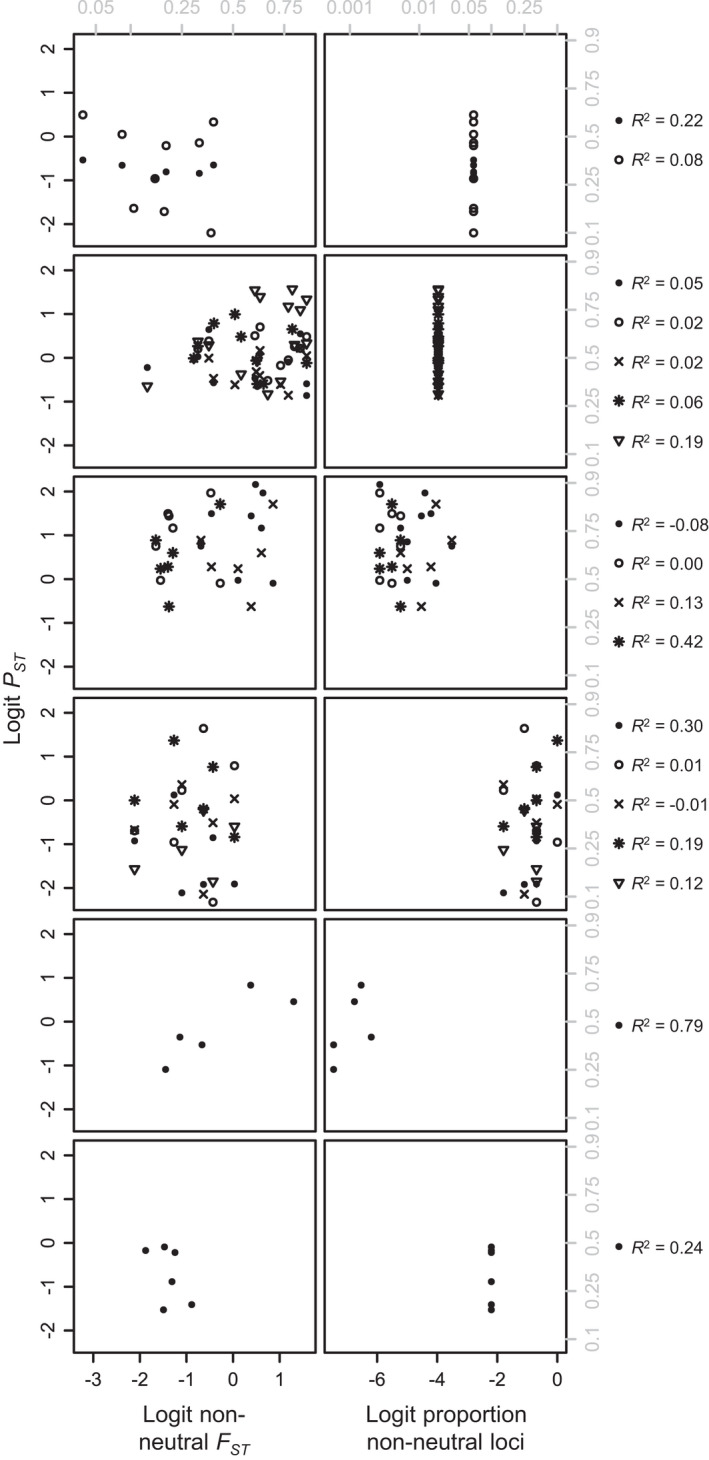
Neither non‐neutral *F*_*ST*_ (*left*) nor proportion of non‐neutral loci (*right*) predicts *P*_*ST*_ consistently well within studies. Each row represents a study; each symbol type represents a phenotype. Data are taken from (top to bottom): Culling et al. (2013), Hamlin & Arnold (2015), Hudson et al. (2013), Kaueffer et al. (2012), Laporte et al. (2015), Raeymaekers et al. (2007). Gray labels show untransformed *F*_*ST*_, *P* *_ST_*, and proportion of non‐neutral loci values. *R* ^2^ values were calculated based on Equation 4, with a few negative values when study‐specific trends in non‐neutral *F*_*ST*_ vs. proportion of non‐neutral loci were the opposite of trends across studies

Within studies, we found no significant relationships between *P*_*ST*_ and *nnF*_*ST*_ for any phenotypes, even with proportion of non‐neutral loci included in the model (Figure 6; Table S2). The mean and standard error for the *P*_*ST*_‐*nnF*_*ST*_ slope within studies were 0.05 and 0.44, respectively, indicating an average *P*_*ST*_‐*nnF*_*ST*_ slope close to zero for individual phenotypes within studies (Figure S4).

## 4 DISCUSSION

### 4.1 Linking genotypic and phenotypic differentiation across and within studies

Here, we show that there is a discernible positive relationship between metrics that measure genomic and phenotypic differentiation when applied to loci and traits putatively under selection (Figure 3). In spite of vast confounding variation within our data set (Table 1)—including species biology, study design, marker type used, statistical approach, and software used—we were able to demonstrate a significant relationship across a diverse range of taxa (i.e., plants, vertebrates, arthropods). This relationship suggests that natural selection acting on the phenome drives evolutionary differentiation on the level of the genome in predictable, somewhat universal ways across clades. The reverse is therefore likely true for the evolutionary processes of drift, mutation, and gene flow, in which genomic change may drive phenotypic change. This work expands on similar findings of congruent genomic and phenotypic differentiation within taxa (Brommer, 2011; Kaeuffer et al., 2012; Raeymaekers et al., 2007).

Our major finding—of a positive relationship between *P*_*ST*_ and non‐neutral *F* *_ST_* (*nnF*_*ST*_) despite the noise of diverse study systems and study designs—has encouraging implications for evolutionary biology. First and foremost, this relationship unsurprisingly supports the genomic basis for phenotypic evolution, suggesting that phenotypic differentiation often has some underlying genomic basis. Of course, this point does not rule out additional environmental contributions like phenotypic plasticity and transgenerational epigenetics. It also suggests that the genomic patterns behind contemporary phenotypic evolution are at least somewhat comparable among taxa on average, even if their characterization is incomplete. Specifically, despite the litany of confounding factors described below, we still found a significant relationship between *P*_*ST*_ and *nnF*_*ST*_, and *nnF*_*ST*_ explained a meaningful proportion of the variation in *P*_*ST*_. With further refinement of genomic methods, standardization and reporting of phenotypic data, and clearer details of how study systems differ in terms of genetic architecture (including the strength of individual loci (many weak vs. few strong), linkage, and gene interactions), this relationship should get clearer.

Practically, our results also suggest that reasonably comparing genomic and phenotypic differentiation across taxa should be possible given standardization of methods and reporting. Such comparisons could prove useful in several situations: first, our results give context for what can be considered large or small differentiation by comparing any given study to the distribution of other studies along the shared axis of genomic and phenomic differentiation presented here (i.e., Figure 4, right panel). Second, the relative phenotypic effect size of a particular genetic locus or set of loci can be captured—at least in part—by looking at the relative size of *P*_*ST*_ and *F*_*ST*_ compared to the values predicted by our model. Finally, as mounting evidence suggests that adaptation in response to selection is generally nonparallel (Bolnick et al., 2018), similar patterns of linked genomic and phenotypic divergence could allow nonparallel adaptation to be compared in terms of degree of differentiation, rather than differentiation in specific traits.

Furthermore, this study contributes to the mounting evidence that contemporary differentiation due to natural selection can provide sufficient, perhaps ideal, phenotypic and genomic variation for linking genomes to phenomes (Evangelou & Ioannidis, 2013). Indeed, the congruence of phenotypic and genomic differentiation across diverse study systems suggests such techniques can be fairly reasonable across taxa. Divergent selection not only produces genomic variation but also generates targeted variation at loci at or near regions that code for responding phenotypes. Thus, differentiation due to natural selection—especially among closely related populations—may generate ideal patterns of genomic variation for genome–phenome association studies by reducing genomic variation at unimportant loci.

### 4.2 Confounding factors

While we have demonstrated a relationship between genomic and phenotypic differentiation, much variation remains, implying the presence of numerous or influential confounding factors. We also acknowledge limitations in our study for identifying these confounding factors; our study only contained data from 31 papers, nor do we have a balanced design covering equal number of papers for each combination of organism, methods, markers, phenotypes, and analysis tool. What follows are our hypotheses for the major sources of variation aside from the genome–phenome mechanism of interest. In general, we believe that variation in the shape of the genomic to phenotypic differentiation relationship comes from three distinct sources: underlying biological, genomic methodological, and phenotypic methodological differences among studies and study systems.

#### 4.2.1 Confounding biological factors

Inherent to the biology of phenotypes are factors that make a universal genome–phenome relationship challenging to elucidate. These factors likely contribute to the problem of missing heritability—that numerous phenotypic traits with quantifiable heritability have genetic underpinnings that remain elusive (Zuk et al., 2012):

1. *Few strong vs*. *many weak loci*. Variation in the strength, number, and interaction of loci underlying phenotypes will affect the nature of the *P*_*ST*_‐*F*_*ST*_ relationship. For example, hundreds of loci likely underlie body size in animals (Kenney‐Hunt et al., 2006), with small changes in many loci (which are challenging to detect) cumulatively leading to large changes in body size. On the other hand, some traits—like stickleback lateral plate number—can be influenced by a few loci of major effect, which will be much easier to detect (Cresko et al., 2007). We would expect the slope of the *P*_*ST*_‐*F*_*ST*_ relationship to be much shallower in the first example compared to the second, even if both phenotypes had an equal additive genetic basis. Furthermore, dominance and epistasis will allow differentiation at some genes to amplify or override differentiation at other genes (Holland, 2007). Developing a *P*_ST_‐*F*_ST_ model that is robust to these variations will require high genomic coverage for *F*_*ST*_ data (to ensure all differentiated loci are found) and methods elucidating gene interactions (Pecanka et al., 2017; Ritchie & Van Steen, 2018).
2. *Genotype–environment interactions*. Environmental effects (intra‐ and transgenerational), including phenotypic plasticity, can also muddle the *P*_*ST*_‐*F*_*ST*_ relationship. Indeed, genotype–environment effects account for a large portion of variation in many phenotypes (Forsman, 2015; Hendry, 2016b). Cogradient plasticity can increase *P*_*ST*_, resulting in an apparently stronger *P*_*ST*_‐*F*_*ST*_ relationship, while countergradient plasticity can decrease *P*_*ST*_, resulting in an apparently weaker *P*_*ST*_‐*F*_*ST*_ relationship (Ghalambor et al., 2007). Plasticity that is unrelated to the gradient in question can still weaken the *P*_*ST*_‐*F*_*ST*_ relationship simply by adding noise to *P*_*ST*_ (Brommer, 2011). Common‐rearing approaches can not only remove plastic effects, but also muddle genetic differentiation in plastic capacity (also known as gene‐by‐environment interactions), thus underestimating *P*_*ST*_. These opposing potential consequences of common‐rearing approaches may explain why common rearing had no significant effect on *P*_*ST*_ or the *P*_*ST*_‐*nnF*_*ST*_ slope in our study (Figure 5). As with many issues in biology, a solution here is to consider results within the context of the specific study organism and examine genetic differentiation, plasticity, and genetic differentiation in plastic capacity (i.e., gene‐by‐environment interactions) through reciprocal‐transplant or multi‐environment common‐rearing studies.

#### 4.2.2 Biases in identifying loci under selection

Different methodological approaches to determining genetic differentiation associated with selection introduce noise to genotype–phenotype relationships between studies. However, as the genome is a relatively concrete feature of an organism, the bias introduced by the choice of genetic and analytical methods can be reduced by systematically identifying appropriate methodology.

1. *Methods used for identifying differentiated loci*. While we found no evidence for favoring any particular method of identifying differentiated loci, we did find a significant *negative* relationship between the proportion of non‐neutral loci identified and *P*_*ST*_ (Figure 4). If most loci had similar phenotypic effect sizes, we would expect a positive relationship between the proportion of non‐neutral loci and *P*_*ST*_, as differentiation that involves more loci of the same phenotypic effect size should generate stronger phenotypic differentiation. We offer two hypotheses as to the observed negative relationship between *P*_*ST*_ and the proportion of non‐neutral loci. The negative relationship may be linked to the effect size of loci (López‐Cortegano & Caballero, 2019). In this case, studies that detected few loci of large effect would have high *P*_*ST*_ values and low proportions of non‐neutral loci, while studies detecting many loci of small effects would have higher proportions of non‐neutral loci, but likely lower *P*_*ST*_, thus generating a negative relationship between the two. Alternatively, the observed negative relationship may instead be linked to methodology, as studies with more liberal classification of loci as non‐neutral (i.e., high false‐positive rates) would report a higher proportion of non‐neutral loci despite relatively low levels of *P*_*ST*_. If this hypothesis is confirmed, more liberal classification of loci as non‐neutral may require down‐weighting of non‐neutral *F*_*ST*_.
2. *Marker choice*. Our results confirm that marker choice induces significant variation into the *nnF_ST_*‐*P_ST_* relationship. While marker choice did not significantly affect the *P*_*ST*_‐*nnF*_*ST*_ slope, including an effect of marker choice on *P*_*ST*_ raised the model *R* ^2^ from 0.25 to 0.39. This apparent effect may be due to the correlation of marker type and genomic coverage, as high genomic coverage (e.g., SNPs) resulted in a lower value of *P*_*ST*_ for a given value of *nnF*_*ST*_ than low genomic coverage (e.g., AFLPs). This result suggests that historical low‐coverage approaches associated with certain marker types may have underestimated *nnF*_*ST*_, leading to higher *P*_*ST*_ values for a given value of *nnF*_*ST*_ (or more intuitively, lower *nnF*_*ST*_ estimates for a given value of *P*_*ST*_).
3. *Estimating F*_*ST*_. While our results indicated no particular best *F*_*ST*_ estimation method in terms of linking *P*_*ST*_ to *nnF*_*ST*_, we note that our observed *P*_*ST*_‐*nnF*_*ST*_ relationship is almost certainly muddied by noise generated by varying software and software settings used to estimate *F*_*ST*_.

#### 4.2.3 Bias in measuring phenotype

Unlike the genome, which has an objective, finite definition as a nucleotide sequence, the “phenome” is inherently subjective (Box 1). Though the phenotype is the object of selection and a physical property determined in part by the genome, different phenotypes must be recognized and defined on a case‐by‐case basis, and it is unlikely that the simple metrics used by researchers to define traits fully capture the more complex integrated phenotypes that are truly under selection. Moreover, investigators may be inconsistent in how they capture traits from study to study. Therefore, we recommend reducing the subjectivity of phenotypic data by standardizing the measurement of traits within taxonomic groups and by capturing a wider array of phenotypes within studies:

1. *Data standardization*. Variable methods for measuring complex phenotypes can make comparisons across studies challenging. For example, features like “body shape” may be quantified several different ways even within a particular clade. As relevant phenotypes are study‐dependent, some variability in measurements is to be expected. However, it is crucial that authors report methods detailing their phenotyping protocols so that phenotypic data that are comparable across studies can be more easily extracted. Even methods for calculating *P*_*ST*_ vary, seemingly arbitrarily, from paper to paper, often without reporting of assumed values of some variables included in calculations (such as heritability). Some disciplines, like macroecology and studies of vertebrate museum specimens, utilize simplified, standardized phenotypic measurements for a particular taxon (Schneider et al., 2019). Adopting similar protocols across and within study systems will increase the power of meta‐analyses and allow for broader comparisons of phenotypic differentiation. For example, standardized descriptions and databases of mutant phenotypes have been developed for a few model taxa (Bogue et al., 2018; Davis et al., 2012; Smith et al., 2004). We also encourage researchers within certain study systems to explore correlative statistical approaches—like structural equation modeling—to describe the relationship among particular phenotypic measurements and how they might relate to the latent phenotypic trait of interest (e.g., body shape). Large strides have been made in behavioral ecology with these methods to understand what phenotypic traits are being measured by different quantifications of behavior (Dingemanse et al., 2010), and similar approaches should be possible for any complex phenotypic trait.
2. *More extensive phenotypic data collection*. Many phenotypes are highly plastic (Forsman, 2015), and detecting causative loci even for those phenotypes with a strong genetic basis may be difficult with reduced‐representation genome‐sequencing methods. Therefore, much as increasing the completeness of genome sequencing increases the chance of finding differentiated loci when they are present, expanding phenomic coverage by measuring more biologically relevant phenotypes increases the chance of finding phenotypes with a strong genetic basis (but, also like genomic methods, requires appropriate multiple comparison statistical corrections). Furthermore, expanded coverage of the phenome will provide additional useful information like an estimate of the background neutral phenotypic differentiation and correlations in degree of differentiation among suites of differentiated traits, both of which could increase the power of association studies. Finally, the more traits measured, the more likely that multivariate phenotypes can be identified that come closer to the latent, integrated phenotypes under selection.

### 4.3 Data archiving

Standardization of data collection and publication methods is necessary to ensure reproducibility and to allow more broad‐scale analyses of the genome‐to‐phenome association like we have done here. Thanks to increasingly common data sharing requirements by journals and broadly standardized data archiving efforts such as GenBank or Dryad, genotypic data are widely available; however, the relatively modest number of studies analyzed here reflects that only a small proportion of papers adequately publish associated phenotypic data. Perhaps the best resource is the Database of Genotypes and Phenotypes (dbGaP) that is used by the human genetics community (Tryka et al., 2014). Going forward, it is imperative that authors ensure relevant phenotypic data and metadata are collected and archived with genotypic data at the time of publication.

Some metadata accessibility issues are common to both genetic and phenotypic data. In particular, thorough metadata and scripts on bioinformatic and analytical pipelines—particularly those including phenotypes (i.e., GWAS)—are often not published in sufficient detail. The inclusion of metadata and workflows for bioinformatic and statistical analyses will improve reproducibility and ensure data are accessible for future analyses as technologies evolve (Broman et al., 2017; Sandve et al., 2013).

### 4.4 Increasing power to link genomes to phenomes

Jarringly, we found no significant relationships between *P*_*ST*_ and *nnF*_*ST*_ for individual phenotypes within studies (Figure 6; Table S2). This result, we hypothesize, suggests that loci identified as differentiated are largely not responsible for the differentiation in phenotypes documented in these studies. This lack of trend is unlikely to be a statistical artifact due to small sample size, as the average intrastudy *P*_*ST*_‐*nnF*_*ST*_ slope was strikingly close to zero (Figure S4). We speculate that a scarcity of population‐level replication may constrain our ability to link genomic differentiation to phenotypic differentiation. Using relatively few populations to identify diverging loci may mask important loci (through lack of variation) and lead us to focus on spurious loci (through random variation).

Based on our ability to infer a general *P*_*ST*_‐*nnF*_*ST*_ relationship across study systems, we suggest several approaches to establishing genome‐to‐phenome relationships within study systems with greater power. These approaches focus on correlating genomic and phenotypic differentiation across metapopulations and involve:

1. Replicate correlations of *P*_*ST*_ and *nnF*_*ST*_ across a multitude of populations spanning a differentiation spectrum.
2. Broad coverage of phenotype (to correspond with broad genomic coverage), including sampling of as many biologically relevant and evolutionarily independent phenotypes as feasible.
3. A standardization of traits documented within taxonomic groups.

Correlating *P*_*ST*_ and *nnF*_*ST*_ across a spectrum of differentiation ensures that genotypes and phenotypes are not only associated, but clearly *differentiate congruently* across landscapes, providing more thorough evidence for the genome–phenome functional link. Having a gradient of differentiation (i.e., numerous *nnF*_*ST*_ and *P*_*ST*_ values) avoids potentially spurious genome–phenome relationships generated by cherry‐picking highly differentiated populations, which may be responsible for the observed weak *P*_*ST*_‐*nnF*_*ST*_ relationship within studies. Measuring numerous phenotypes increases the likelihood of finding a phenotype that is strongly determined by diverging loci, as long as appropriate statistical corrections are used to avoid false positives, and also helps us better understand how a given trait diverges in reference to the rest of the phenome. With enough careful measurement, we should be able to describe the relationship between *nnF*_*ST*_ and *nnP*_*ST*_, the non‐neutral components of phenotype. Currently, our only approach to this is to use our limited understanding of the system to select what we judge as the most differentiated traits. Through careful methodological choices, broader measurement of phenotypes, and a metapopulation approach, genome‐to‐phenome associations in natural populations can become a more powerful and accessible tool for understanding contemporary evolution.

## CONFLICT OF INTEREST

The authors declare no conflicts of interest.

## AUTHOR CONTRIBUTION

Project design: All authors. Project funding: BJO, BLK, AIK, MTK. Literature search: AKW, ZTW. Data collection: ZTW, AKW, KLB, JDC. Analysis: ZTW. Figure making: ZTW, AKW, KLB, JDC. Lead writing: ZTW, AKW, KLB, JDC, JJH. Editing and revision: All authors.

## Supporting information

Supplementary Material

Supplementary Material

## Data Availability

The data that support the findings of this study are available in the supplementary material of this article.
