# Supplementary material for: Meta‐analysis: Congruence of genomic and phenotypic differentiation across diverse natural study systems
Source: Evol Appl. 2021 Aug 19;14(9):2189–205. doi: 10.1111/eva.13264 (PMC8477602; doi:10.1111/eva.13264)
Supplement: Supplementary file 1 — Supplementary Material [file EVA-14-2189-s002.pdf]

SUPPLEMENT

CONGRUENCE OF GENOMIC AND PHENOTYPIC DIFFERENTIATION ACROSS DIVERSE WILD STUDY SYSTEMS

Table S-1. Search terms used to search all databases across all years within Web of Science on July 17th 2019.

**Returned >700 or 0 results; not used**	**Returned <700 results; used**
Genome morphology	Bayescan morphology
Genome phenotype	Bayescan phenotype
Genome PST	Bayescan PST
GWAS FST divergence PST	Candidate FST divergence morphology
Lositan PST	Candidate FST divergence phenotype
Sequencing morphology	Candidate FST divergence PST
Sequencing phenotype	Divergent selection morphology genotype
Sequencing PST	GWAS FST divergence morphology
SNP morphology	GWAS FST divergence phenotype
SNP phenotype	Lositan morphology
	Lositan phenotype
	Outlier FST divergence morphology
	Outlier FST divergence phenotype
	Outlier FST divergence PST
	SNP PST

Table S-2. Parameter estimates and significance tests for P_{ST}-nnF_{ST} relationships for individual phenotypes within papers. See Equation 4 for model formulation. β_L = 0.13 ± 0.19

Paper	Trait	Method	β_Z		β_{ZF}			
			est.	s.e.	est.	s.e.	t	p
Culling et al 2013	Parr mark contrast	FSTAT	-0.77	0.74	-0.29	0.29	-1.02	0.308
	Parr mark value		-0.50	0.82	-0.07	0.33	-0.21	0.833
Hamlin et al 2015	Leaf height	Bayescan	0.73	0.81	-0.17	0.31	-0.53	0.597
	Leaf width		0.75	0.80	0.37	0.21	1.79	0.077
	Nectar guide		0.18	0.83	0.07	0.35	0.21	0.837
	Petal length		0.34	0.80	-0.09	0.20	-0.44	0.657
	Petal width		0.75	0.84	-0.06	0.32	-0.19	0.851
Hudson et al 2013	Gill Raker Number	Bayescan	1.68	1.33	0.01	0.57	0.02	0.980
		DFDIST	1.65	0.93	0.16	0.45	0.37	0.714
	LM PC1	Bayescan	2.51	1.41	1.02	0.68	1.51	0.134
		DFDIST	1.03	0.90	0.31	0.57	0.56	0.580
Kaeuffer et al 2012	Carbon Isotope	Conf. Int.	0.29	0.55	0.44	0.45	0.96	0.337
	Plate number		-0.66	0.55	0.33	0.47	0.72	0.473
	Proportion limnetic prey		-1.59	0.55	-0.47	0.45	-1.04	0.302
	Relative warp 1		-0.08	0.55	-0.30	0.45	-0.65	0.515
	Trophic position		-0.41	0.55	-0.01	0.45	-0.03	0.979
Laporte et al 2015	Multiple ANOVA	FDIST	0.92	1.37	0.57	0.33	1.70	0.093
Raeymaekers et al 2007	Distal short gill raker number	DETSEL	-1.79	1.53	-0.98	1.04	-0.94	0.348

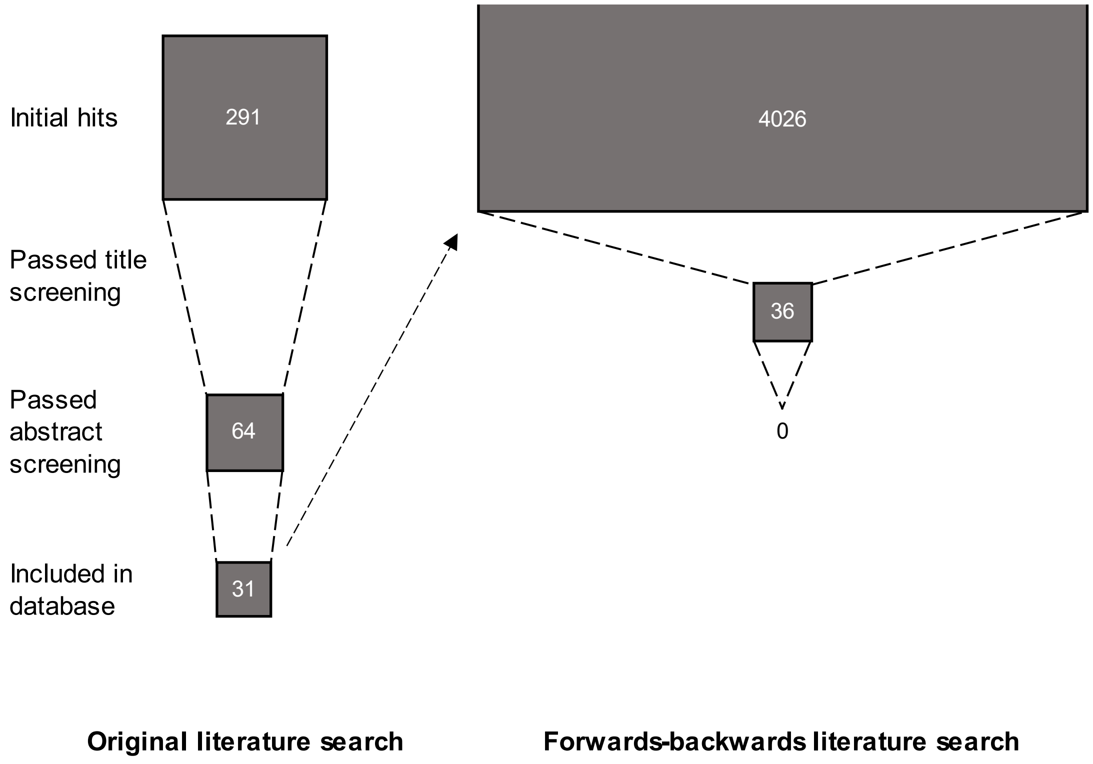

Figure S-1. Numbers of papers screened at each level of our literature search. Note that there was no title screening during the original literature search.

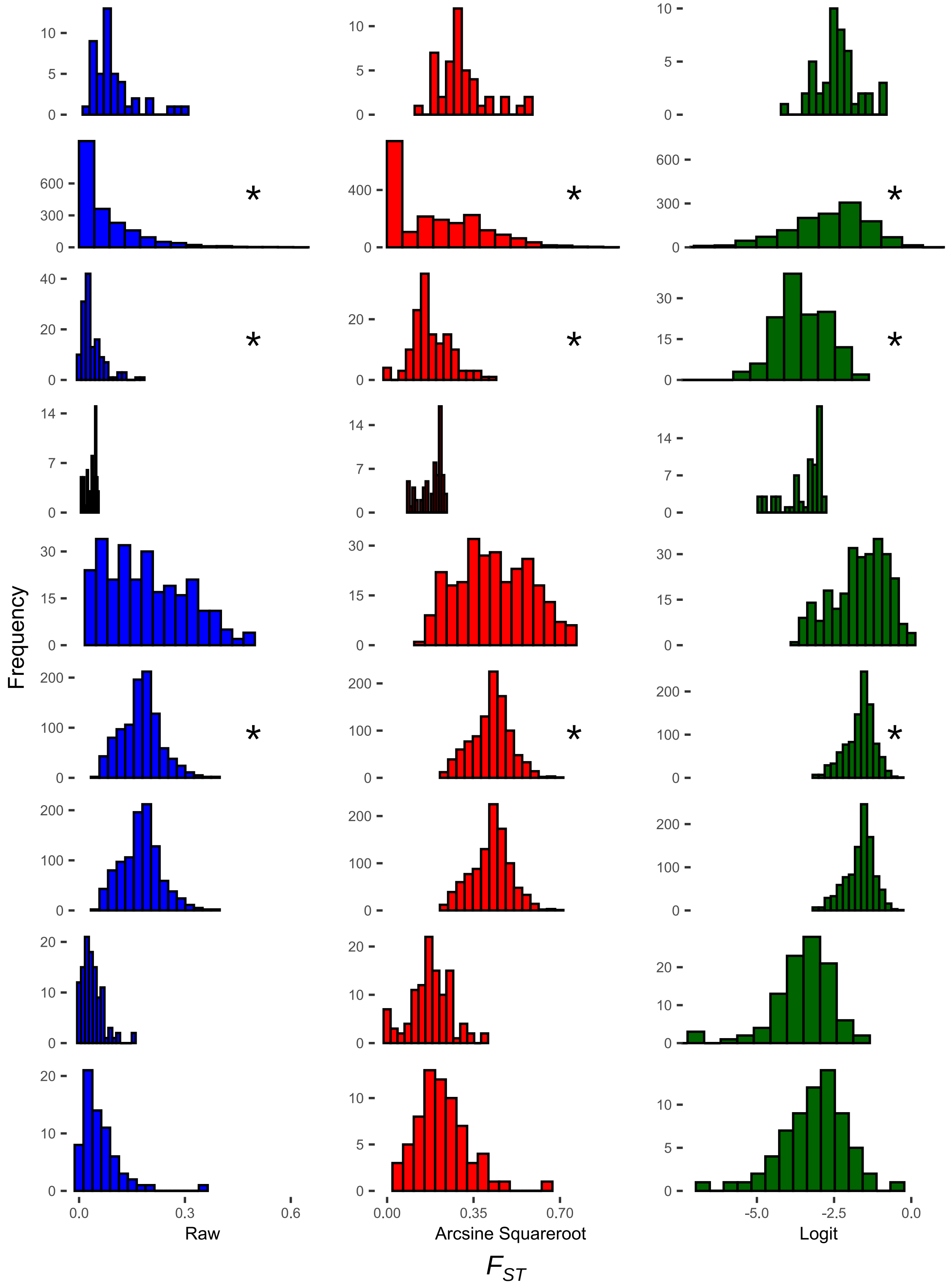

Figure S-2. Arcsine square root transformation, or, preferably, logit transformation of F_{ST} data visually reduce skew and improve normality. Data shown are from Ackerman et al., 2011; Black et al., 2017; Defaveri and Merilä, 2013; Flanagan et al., 2016; Hamilton et al., 2013; Porth et al., 2015; Song et al., 2016; Whiteley et al., 2011, and include all loci, regardless of neutrality. * indicates studies for which we changed all F_{ST} values < 0.001 to 0.001. Negative F_{ST} values are biologically unrealistic and cannot be arcsine square root or logit transformed.

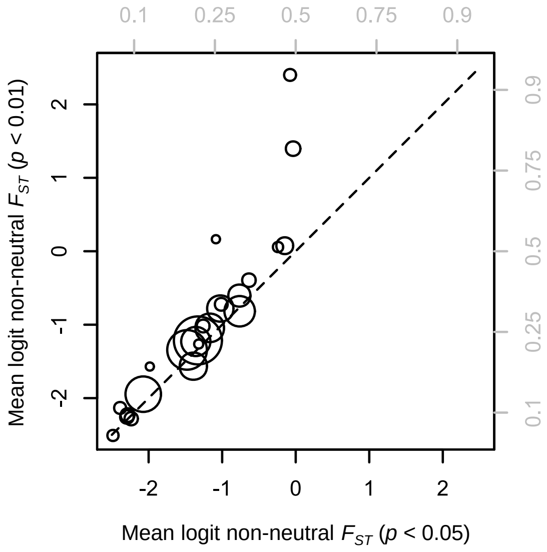

Figure S-3. Average outlier F_{ST} for two different *p*-value thresholds. Average logit outlier F_{ST} is only weakly sensitive to changing the *p* threshold from 0.05 to 0.01, with $r = 0.90$. Each point here represents a unique population-population comparison for which outliers at both $p < 0.05$ and $p < 0.01$ are reported. Point size here indicates number of loci for $p < 0.05$, which range from 2 to 2402. Data here are from Black et al., 2017; Chen et al., 2013; Culling et al., 2013; D'Anatro, 2017; Defaveri and Merilä, 2013; Hemond and Wilbur, 2011; Kovi et al., 2015; Lv et al., 2014; Mastrangelo et al., 2019; Nichols et al., 2016; Qiu et al., 2017; Roschanski et al., 2016; Tomicic et al., 2015.

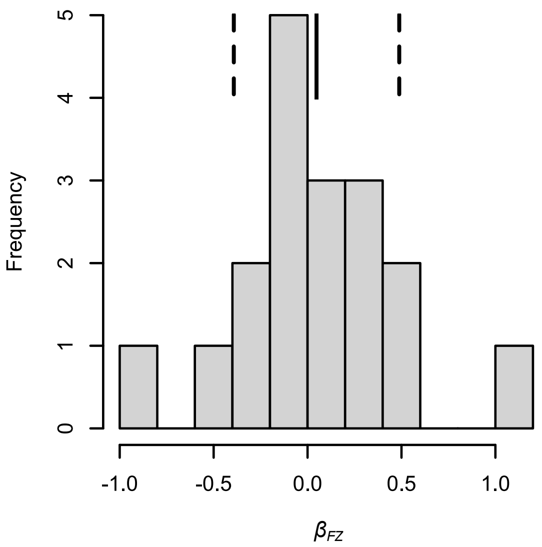

Figure S-4. Histogram of glm-estimated P_{ST}-nnF_{ST} slopes for individual phenotypes within papers. Solid and dashed vertical lines show mean ± standard error, respectively. See Table S-2 for individual slope estimates and significance tests.

References

Ackerman, M.W., Habicht, C., and Seeb, L.W. (2011). Single-nucleotide polymorphisms (SNPs) under diversifying selection provide increased accuracy and precision in mixed-stock analyses of sockeye salmon from the Copper River, Alaska. Trans. Am. Fish. Soc. *140*, 865–881.

Black, A.N., Seears, H.A., Hollenbeck, C.M., and Samollow, P.B. (2017). Rapid genetic and morphologic divergence between captive and wild populations of the endangered Leon Springs pupfish, Cyprinodon bovinus. Mol. Ecol. *26*, 2237–2256.

Chen, S., Gomes, R., Costa, V., Santos, P.C. dos, Charneca, R., Zhang, Y., Liu, X., Wang, S., Bento, P., Nunes, J.-L., et al. (2013). How immunogenetically different are domestic pigs from wild boars: a perspective from single-nucleotide polymorphisms of 19 immunity-related candidate genes. Immunogenetics *65*, 737–748.

Culling, M.A., Freamo, H., Patterson, K.P., Berg, P.R., Lien, S., and Boulding, E.G. (2013). Signatures of selection on growth, shape, parr marks, and SNPs among seven canadian Atlantic salmon (Salmo salar) populations. p.

D'Anatro, A. (2017). Correlation between environmental factors, a life history trait, phenotypic cohesion, and gene flow levels in natural populations of Micropogonias furnieri: is salinity the main factor driving divergence? Estuaries Coasts *40*, 1717–1731.

Defaveri, J., and Merilä, J. (2013). Evidence for adaptive phenotypic differentiation in Baltic Sea sticklebacks. J. Evol. Biol. *26*, 1700–1715.

Flanagan, S.P., Rose, E., and Jones, A.G. (2016). Population genomics reveals multiple drivers of population differentiation in a sex-role-reversed pipefish. Mol. Ecol. *25*, 5043–5072.

Hamilton, J.A., Lexer, C., and Aitken, S.N. (2013). Differential introgression reveals candidate genes for selection across a spruce (Picea sitchensis × P. glauca) hybrid zone. New Phytol. *197*, 927–938.

Hemond, E.M., and Wilbur, A.E. (2011). Microsatellite loci indicate population structure and selection between Atlantic and Gulf of Mexico populations of the bay scallop Argopecten irradians. Mar. Ecol. Prog. Ser. *423*, 131–142.

Kovi, M.R., Fjellheim, S., Sandve, S.R., Larsen, A., Rudi, H., Asp, T., Kent, M.P., and Rognli, O.A. (2015). Population structure, genetic variation, and linkage disequilibrium in perennial ryegrass populations divergently selected for freezing tolerance. Front. Plant Sci. *6*.

Lv, F.-H., Agha, S., Kantanen, J., Colli, L., Stucki, S., Kijas, J.W., Joost, S., Li, M.-H., and Ajmone Marsan, P. (2014). Adaptations to climate-mediated selective pressures in sheep. Mol. Biol. Evol. *31*, 3324–3343.

Mastrangelo, S., Bahbahani, H., Moioli, B., Ahbara, A., Abri, M.A., Almathen, F., Silva, A. da, Belabdi, I., Portolano, B., Mwacharo, J.M., et al. (2019). Novel and known signals of selection for fat deposition in domestic sheep breeds from Africa and Eurasia. PLOS ONE *14*, e0209632.

Nichols, K.M., Kozfkay, C.C., and Narum, S.R. (2016). Genomic signatures among Oncorhynchus nerka ecotypes to inform conservation and management of endangered Sockeye Salmon. Evol. Appl. *9*, 1285–1300.

Porth, I., Klápště, J., McKown, A.D., Mantia, J.L., Guy, R.D., Ingvarsson, P.K., Hamelin, R., Mansfield, S.D., Ehlting, J., Douglas, C.J., et al. (2015). Evolutionary quantitative genomics of Populus trichocarpa. PLOS ONE *10*, e0142864.

Qiu, T., Jiang, L., Li, S., and Yang, Y. (2017). Small-scale habitat-specific variation and adaptive divergence of photosynthetic pigments in different alkali soils in reed identified by common garden and genetic tests. Front. Plant Sci. *7*.

Roschanski, A.M., Csilléry, K., Liepelt, S., Oddou-Muratorio, S., Ziegenhagen, B., Huard, F., Ullrich, K.K., Postolache, D., Vendramin, G.G., and Fady, B. (2016). Evidence of divergent selection for drought and cold tolerance at landscape and local scales in Abies alba Mill. in the French Mediterranean Alps. Mol. Ecol. *25*, 776–794.

Song, Z., Zhang, M., Li, F., Weng, Q., Zhou, C., Li, M., Li, J., Huang, H., Mo, X., and Gan, S. (2016). Genome scans for divergent selection in natural populations of the widespread hardwood species Eucalyptus grandis (Myrtaceae) using microsatellites. Sci. Rep. *6*, 1–13.

Tomicic, M., Djordjevic, V., Obreht, D., Miladinovic, J., Brbaklic, L., Mikic, A., and Mikic, S. (2015). Tracking footprints of selection associated with soybean adaptation to Central-East Europe environments. Euphytica *203*, 701–713.

Whiteley, A.R., Bhat, A., Martins, E.P., Mayden, R.L., Arunachalam, M., Uusi-Heikkilä, S., Ahmed, A.T.A., Shrestha, J., Clark, M., Stemple, D., et al. (2011). Population genomics of wild and laboratory zebrafish (Danio rerio). Mol. Ecol. *20*, 4259–4276.
